# Delphi-Based Consensus on Interstitial Lung Disease Screening in Patients with Connective Tissue Diseases (Croatian National-Based Study)

**DOI:** 10.3390/biomedicines10123291

**Published:** 2022-12-19

**Authors:** Mislav Radić, Srđan Novak, Marko Barešić, Ana Hećimović, Dijana Perković, Jasna Tekavec-Trkanjec, Miroslav Mayer, Višnja Prus, Jadranka Morović-Vergles, Daniela Marasović Krstulović, Mislav Cerovec, Ljiljana Bulat Kardum, Miroslav Samaržija, Branimir Anić

**Affiliations:** 1 Division of Rheumatology and Clinical Immunology, Centre of Excellence for Systemic Sclerosis Ministry of Health Republic of Croatia, University Hospital Centre Split, 21000 Split, Croatia; 2 School of Medicine, University of Split, 21000 Split, Croatia; 3 Department of Rheumatology and Clinical Immunology, University Hospital Center Rijeka, 51000 Rijeka, Croatia; 4 School of Medicine, University of Rijeka, 51000 Rijeka, Croatia; 5 Division of Clinical Immunology and Rheumatology, Department of Internal Medicine, University Hospital Center Zagreb, 10000 Zagreb, Croatia; 6 School of Medicine, University of Zagreb, 10000 Zagreb, Croatia; 7 Clinic for Respiratory Diseases, University Hospital Center Zagreb, 10000 Zagreb, Croatia; 8 Department of Pneumonology, Dubrava University Hospital, 10000 Zagreb, Croatia; 9 Department of Rheumatology, Clinical Immunology and Allergology, University Hospital Center Osijek, 31000 Osijek, Croatia; 10 School of Medicine, University of Osijek, 31000 Osijek, Croatia; 11 Division of Clinical Immunology, Allergology and Rheumatology, Department of Internal Medicine, Dubrava University Hospital, 10000 Zagreb, Croatia; 12 Department for Rheumatology, Special Hospital Primamed, 10000 Zagreb, Croatia; 13 Department of Pneumonology, University Hospital Center Rijeka, 51000 Rijeka, Croatia

**Keywords:** interstitial lung disease, systemic sclerosis, mixed connective tissue disease, systemic lupus erythematosus, Sjögren’s syndrome, rheumatoid arthritis, idiopathic inflammatory myopathy, national delphi consensus

## Abstract

The aim of this study was to develop a Croatian Delphi-based expert consensus for screening interstitial lung disease (ILD) associated with connective tissue disease (CTD). A systematic literature review was conducted on risk factors for the development of ILD, prevalence and incidence of ILD, diagnostic and screening methods for ILD, and prognosis of ILD in idiopathic inflammatory myopathy (IIM), mixed connective tissue disease (MCTD), primary Sjögren’s syndrome (pSS), rheumatoid arthritis (RA), systemic lupus erythematosus (SLE), and systemic sclerosis (SSc) were performed. Based on the evidence found, experts developed questionnaires for screening and monitoring ILD in each CTD, which were provided via an online survey. Following the electronic survey, two screening algorithms were developed based on the consensus opinions. The detection strategy for ILD included high-resolution computed tomography (HRCT) in addition to pulmonary function testing for IIM, MCTD, and SSc. and pulmonary function testing for newly diagnosed pSS, RA and SLE. However, in patients with identified risk factors for ILD HRCT, these tests should also be performed. A screening strategy for early identification of patients with various CTD-ILD was first developed by a multidisciplinary team of rheumatologists, pulmonologists, and radiologists to identify early CTD patients at risk of ILD, a severe extra-articular manifestation of CTD.

## 1. Introduction

Connective tissue diseases (CTDs) are a heterogeneous group of inflammatory disorders affecting many systems and organs. The respiratory system is particularly susceptible to autoimmune-mediated chronic inflammation, as well as the adverse effects of CTD treatments. Multiple components of the respiratory system may be affected: airway, vascular, parenchyma, pleura, and respiratory musculature. Interstitial lung disease (ILD) is a major cause of morbidity and mortality in CTD. Although ILD is usually a late manifestation of CTD, the time of onset varies for CTD-ILD. ILD has been observed in patients years before the onset of CTD, and 30% to 60% of patients have radiographic evidence of subclinical ILD [1]. Pulmonary function tests and high-resolution computed tomography (HRCT) are useful tools for the screening and early detection of CTD-ILD. ILD can occur in all types of CTDs, and regular use of HRCT increases the detection of pulmonary involvement. The prevalence of ILD varies among CTDs and is considered to be highest in systemic sclerosis (SSc) and lowest in systemic lupus erythematosus (SLE) [2].

The reported prevalence of ILD in specific CTDs varies from study to study, partly because of different measures (i.e., point prevalence, annual prevalence, or other) and geographic regions, and partly because of diagnostic procedures. Considering studies based on HRCT: (i) in European SSc patients, the estimated prevalence of ILD is 19–47% [3], (ii) the prevalence in mixed connective tissue disease (MCTD) is estimated to be about 40% [4], (iii) ILD is reported to be found in up to 60% of patients with rheumatoid arthritis (RA) and about 10% of RA patients develop clinically significant ILD [5,6,7], (iv) the prevalence of ILD in patients with idiopathic inflammatory myopathy (IIM) (mostly polymyositis/dermatomyositis) is estimated to be 41% [8], (v) the prevalence of ILD in primary Sjoegren’s syndrome (pSS) is about 20% [9], and (vi) the prevalence of ILD associated with SLE-related ILD is estimated to be 3% to 13% [7].

There is a growing awareness of the incidence/prevalence of ILD in CTDs, the specifics of ILD associated with various underlying CTDs, and the need for timely (early) diagnosis of ILD to maximize the benefits of treatment. Due to the diversity of existing viewpoints and practices and the concurrent lack of uniform and straightforward guidelines/recommendations, we aimed to develop a national (Croatian) expert consensus for the diagnosis and follow-up of CTD-ILD in IIM, MCTD, pSS, RA, SLE, and SSc. These recommendations should focus on the process starting with newly diagnosed CTD patients, the need for initial screening for ILD, and further procedures during the course of the disease to detect later developing ILD early.

## 2. Materials and Methods

A panel of experts was formed from 14 Croatian specialists in clinical immunology/rheumatology (*n* = 10), pulmonology (*n* = 3), and radiology (*n* = 1), who were selected on the basis of their interest in and experience with the topic under discussion and their scientific merit. Because the objective/topic was fixed a priori, the Delphi procedure performed consisted of four steps. 1. In the first session, the experts discussed and agreed on the following: (i) one of the experts (MR) was appointed moderator of the process; (ii) literature databases (Medline, Embase, Cochrane Library) were to be searched to identify (and subsequently rescreen) relevant publications on epidemiological aspects (prevalence, incidence, risk factors) of ILD and on screening, diagnosis, and monitoring methods for ILD specifically in individual CTDs; (iii) given disease (dis)similarities, some CTDs could receive common recommendations while others should be addressed individually, specifically MCTD and SSc should be addressed jointly, SLE and pSS should be addressed jointly, while RA and IIM should be addressed individually; (iv) for the voting process, defining points in terms of multiple response questions is preferred to defining points in terms of statements with Likert scale responses. Answers where at least 80% agreement is achieved (i.e., at least 11/14 votes) would be considered “agreed”. Items for which no agreement is reached after 2 rounds of voting would be resolved by direct discussion; (v) four groups of 3–4 members each were formed to define a questionnaire on one of the four disease groups (MCTD + SSc, pSS + SLE, RA, IIM) based on the reviewed literature and (cumulative) clinical experience. 2. After 5 weeks, a second meeting was held to present the defined questionnaires, discuss their format and content, and modify them if necessary. 3. Anonymous voting was completed via an online survey. 4. At the follow-up meeting about a month after the voting, consensual recommendations and illustrative flowcharts were created based on the voting results.

## 3. Results

All clinical immunology/rheumatology, pulmonology, and radiology experts (*n* = 14) invited to participate in the Delphi survey provided their responses via an electronic survey. Consensus responses of involved experts for the screening and monitoring of ILD in the six CTDs are provided in Table 1, Table 2, Table 3 and Table 4.

Individual risk factors and associations for CTD-ILD such as age, gender, smoking, reflux, disease activity, gene studies, and serological status were discussed at the second meeting. Clinical judgment of the treating physician was based on the signs and symptoms of ILD, but as patients are often asymptomatic, risk factors associated with ILD-CTD were also taken into consideration. The most common symptoms of ILD-CTD include shortness of breath, exertional dyspnea, dry cough without phlegm and weakness/extreme fatigue. Other symptoms include loss of appetite, unexplained weight loss, chest discomfort, dyspnea, tachypnea, and pulmonary hemorrhage. Susceptibility genes and autoantibodies and serological biomarkers testing associated with ILD-CTD (Table 5) are routinely available at tertiary centers; however, they are not readily available at smaller treating centers due to development of the healthcare system and economics.

**Figure 1:**
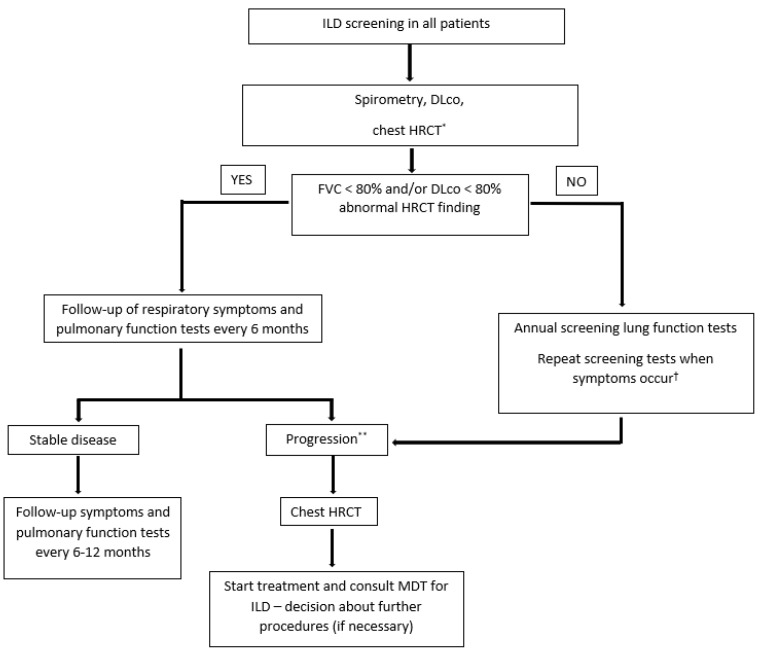
Screening for ILD in newly discovered MCTD, IIM, or SSc patients. Abbreviations: DLco, diffusion capacity for carbon monoxide; FVC, forced vital capacity; HRCT, high-resolution computed tomography; IIM, idiopathic inflammatory myopathy; ILD, interstitial lung disease; MCTD, mixed connective tissue disease; MDT; NSIP; SSc, systemic sclerosis. * Especially in patients with antisynthetase positive antibodies and anti-MDA5 in idiopathic inflammatory myopathy and anti-Scl-70/anti-topoisomerase I antibody in SSc. ** Clinical deterioration and/or decrease in FVC or DLco below 80% or by more than 10% compared to previous findings. ^†^ Most common symptoms include shortness of breath, exertional dyspnea, dry cough without phlegm, and weakness/extreme fatigue. Other symptoms include loss of appetite, unexplained weight loss, chest discomfort, dyspnea, tachypnea, and pulmonary hemorrhage.

Following the Delphi survey, a screening algorithm was generated based on the consensus opinions for ILD in newly discovered MCTD, IIM, and SSc patients was developed (Figure 1).

Pulmonary function tests should be performed in all newly diagnosed patients. Presence of certain disease specific antibodies merits close monitoring and inclusion of chest HRCT as a screening method as these antibodies have been associated with the clinically significant development of pulmonary fibrosis. Patients with no abnormalities should be monitored annually and in case of ILD, repeated pulmonary function tests should be performed every 6 months. Disease progression is defined as clinical deterioration and/or decrease in forced vital capacity (FVC) or diffusion capacity for carbon monoxide (DLco) below 80% or by more than 10% compared to previous findings. Progressive disease should be monitored by chest HRCT and managed. A multidisciplinary team should be consulted if deemed necessary by the treating physician.

**Figure 2:**
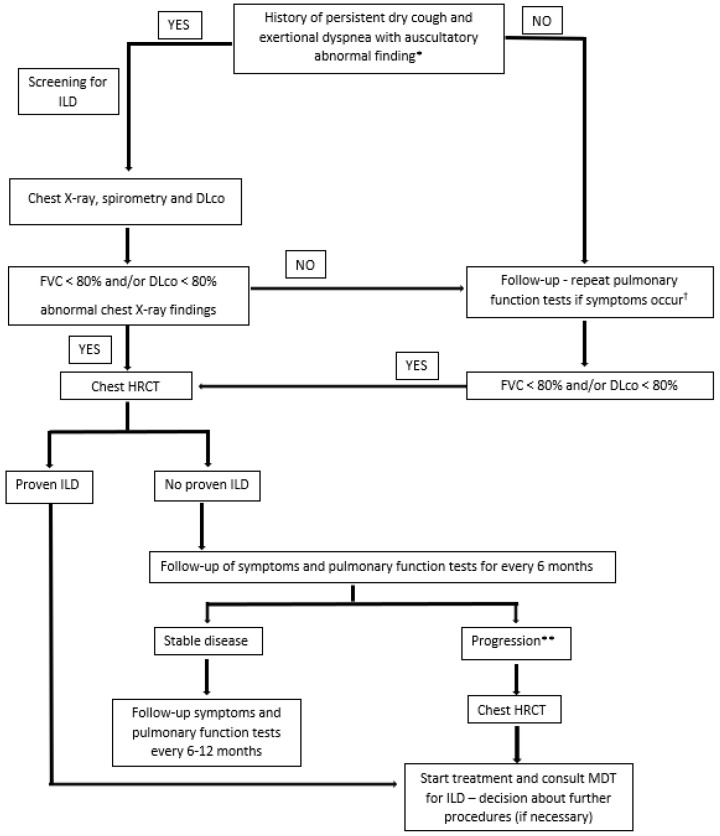
Screening for ILD in newly discovered RA, pSS, or SLE patients. Abbreviations: DLco, diffusion capacity for carbon monoxide; FVC, forced vital capacity; ILD, interstitial lung disease; HRCT, high-resolution computed tomography; MDT; multidisciplinary team; pSS, primary Sjögren’s syndrome; RA, rheumatoid arthritis; SLE, systemic lupus erythematosus. * In patients with identified risk factors include HRCT as screening methods. ** Clinical deterioration and/or decrease in FVC or DLco below 80% or by more than chest x compared to previous findings. ^†^ Most common symptoms include shortness of breath, exertional dyspnea, dry cough without phlegm, and weakness/extreme fatigue. Other symptoms include loss of appetite, unexplained weight loss, chest discomfort, dyspnea, tachypnea, and pulmonary hemorrhage.

An additional screening algorithm was generated based on expert consensus for ILD in newly discovered pSS, RA, and SLE patients (Figure 2).

Patients with pulmonary signs and symptoms should be referred to for ILD screening (chest X-ray, spirometry and DLco). The presence of risk factors merits the inclusion of chest HRCT at the time of the initial presentation. Chest X-ray has low sensitivity and specificity for establishing diagnosis of CTD-ILD but can be helpful in identifying patients with pulmonary manifestations of CTD. Most common radiologic findings that can be found are reticular or reticulonodular opacities. In the case of these findings, patients should be further referred for HRCT. Patients with no abnormalities should be referred for a pulmonary function test in case of symptom worsening. Patients with proven ILD on HRCT should be followed-up every 6 months. Progressive disease should be monitored by chest HRCT and managed. A multidisciplinary team should be consulted if deemed necessary by the treating physician.

## 4. Discussion

The goal of our Delphi-based consensus was to improve the early detection of ILD in CTD and the follow-up of these patients. ILD in CTD can be detected at any time point and varies in the type of ILD and severity. The reported prevalence of ILD in patients with CTD varies according to classification criteria and study registries for specific diagnoses, with a higher frequency in IIM, MCTD, and SSc and a lower frequency in SLE [33]. The prevalence of ILD in different CTDs and its impact on prognosis varies considerably, so we make our recommendations for the higher frequency group ILD IIM, MCTD, and SSc and the lower frequency group pSS, RA, and SLE [10]. It is important to consider that in CTD-ILD, the progression of fibrosis is slower than in idiopathic pulmonary fibrosis and the extent of pulmonary fibrosis on the first HRCT is lower [34]. Fibrosing nonspecific interstitial pneumonia is the most common pattern identified in most CTD, while ordinary interstitial pneumonia (UIP) is the most common form of RA-ILD [10]. SSc presents as cellular bronchiolitis separately or in association with fibrosing nonspecific interstitial pneumonia, while RA presents as follicular bronchiolitis and occasionally as organizing pneumonia [35,36]. Although in some published data the prevalence of RA-ILD is as high as 60%, only a minority of these patients develop clinically significant disease [5,6,7]. Experts have agreed that the low incidence of clinically relevant RA-ILD in their practice does not justify its inclusion in the group of diseases with a high prevalence of CTD-ILD, although it should be noted that the symptoms of RA are very similar to idiopathic pulmonary fibrosis and predict worse survival [7]. The unmet need for a screening strategy for ILD in RA was recently addressed by a Spanish multidisciplinary team [37]. It is important to point out that we should be careful to exclude diffuse alveolar hemorrhage in patients with SLE who develop acute respiratory symptoms and respiratory failure.

In addition, there is a wide spectrum of histopathologic patterns within each CTD [38]. Because of the differences in the prevalence of clinically relevant manifestations and histopathologic patterns of ILD in different CTDs, the diseases have been grouped and algorithms developed for screening for ILD in newly detected MCTD, IIM, or SSc patients/screening for ILD in newly detected pSS, RA, or SLE patients.

Pulmonary symptoms also affect patients’ quality of life and early detection of CTD-ILD is important for deciding on a change in treatment strategy [39,40]. If symptoms worsen, lung function deteriorates, and findings on HRCT progress during follow-up, it is important to rule out other causes such as infection or drug-induced lung disease. In these cases, patients should be discussed by the multidisciplinary ILD team to determine whether further investigations such as bronchoscopy or surgical biopsy are needed. This Delphi consensus has confirmed that HRCT is an important tool to detect and characterize pulmonary pathology, particularly ILD, in CTD patients. The pulmonary function test is recommended to assess the severity of the diseases and to support the decision to initiate treatment and predict prognosis [40]. In addition, Delphi-based consensus conclusions strongly support the central role of pulmonary function testing in the evaluation and monitoring of ILD in CTDs.

Although HRCT is the gold standard for ILD screening, there is a need to validate other radiation-free, readily available, noninvasive, bedside screening techniques such as lung ultrasound [41,42]. As more information becomes available, the inclusion of ultrasound in the algorithm will be considered based on the high sensitivity observed to date.

Although HRCT has revolutionized the diagnosis of ILD, interpretation of HRCT of the chest is a complex, challenging process. Therefore, the images should be evaluated by experienced thoracic radiologists, preferably in a center of excellence. The expertise needed to assess the images should therefore be centralized, especially in small countries such as Croatia with a population of approximately 4 million people. Because of the geographic location, images should be sent for centralized evaluation to reduce travel and time to diagnosis.

The above points were considered in the proposed approach for screening patients with IIM, MCTD, and SSc to detect ILD early in these patients before it becomes clinically apparent. Therefore, by applying this algorithm in clinical practice, physicians can diagnose IIM, MCTD, and SSc patients with incidentally found abnormalities on HRCT scans without clear symptoms. Patients with pSS, RA, and SLE should be monitored clinically, and pulmonary function tests should be performed regularly. Experts agreed that if there are clinical findings, HRCT should be performed to identify patients with ILD and facilitate the decision-making process for management and follow-up. However, in patients with identified risk factors, HRCT should also be performed at initial presentation, especially in RA patients. The high level of agreement among experts was considered to be a consequence of the high level of expertise in the relevant field.

## 5. Conclusions

This screening strategy for the early detection of patients with various CTD-ILD was developed for the first time by a multidisciplinary team of rheumatologists, pulmonologists, and radiologists. The aim of the algorithm is to help rheumatologists in the early detection of CTD patients at risk of developing ILD, a severe extra-articular manifestation of CTD.

## Figures and Tables

**Table 1:** Consensus results of the MCTD and SSc questionnaire for screening and monitoring ILD.

Question	Consensus (*n* = 14)
1. Should patients with newly diagnosed MCTD/SSc be referred to initial consultations for ILD screening (HRCT)?	Patients should be referred for ILD screening (*n* = 14)
2. If you answered the previous question with yes, based on clinical judgment which of the following is important/essential in deciding on HRCT?	Pulmonary function tests (*n* = 13) Symptoms, i.e., exertional dyspnea (*n* = 11)
3. If the patient does not have ILD (or other pulmonary pathology) on HRCT, how should possible ILD development be monitored in the patient?	Disease progression should be monitored by HRCT based on clinical judgment (*n* = 12)
4. If you answered HRCT should be repeated based on clinical judgment, which of the following parameters should be taken into consideration when deciding?	Pulmonary function tests (*n* = 10) Symptoms, i.e., new onset of exertional dyspnea (*n* = 10)
5. In a patient with MCTD/SSc and confirmed ILD, should HRCT be repeated during follow-up?	Disease progression should be monitored by HRCT based on clinical judgment (*n* = 12)
6. If you answered HRCT should be repeated based on clinical judgment, which of the following parameters should be taken into consideration when deciding?	Pulmonary function tests (*n* = 11) Symptoms, i.e., new onset of exertional dyspnea (*n* = 9)

Abbreviations: HRCT, high-resolution computed tomography; ILD, interstitial lung disease; MCTD, mixed connective tissue disease; SSc, systemic sclerosis.

**Table 2:** Consensus results of the pSS and SLE questionnaire for screening and monitoring ILD.

Question	Consensus (*n* = 14)
1. Should patients with newly diagnosed pSS/SLE be referred to initial consultations for ILD screening (HRCT)?	Patients should be referred for ILD screening (*n* = 13)
2. If you answered the previous question with yes, based on clinical judgment which of the following is important/essential in deciding on HRCT?	Pulmonary function tests (*n* = 13) Symptoms, i.e., exertional dyspnea or dry cough (*n* = 12)
3. If the patient does not have ILD (or other pulmonary pathology) on HRCT, how should possible ILD development be monitored in the patient?	Disease progression should be monitored by HRCT based on clinical judgment (*n* = 13)
4. If you answered HRCT should be repeated based on clinical judgment, which of the following parameters should be taken into consideration when deciding?	Symptoms, i.e., new onset of dry cough (*n* = 12) Pulmonary function tests (*n* = 10)
5. In a patient with pSS/SLE and confirmed ILD, should HRCT be repeated during follow-up?	Disease progression should be monitored by HRCT based on clinical judgment (*n* = 14)
6. If you answered HRCT should be repeated based on clinical judgment, which of the following parameters should be taken into consideration when deciding?	Pulmonary function tests (*n* = 13) Symptoms, i.e., new onset of exertional dyspnea or dry cough (*n* = 11)

Abbreviations: HRCT, high-resolution computed tomography; ILD, interstitial lung disease; pSS, primary Sjögren’s syndrome; SLE, systemic lupus erythematosus.

**Table 3:** Consensus results of the RA questionnaire for screening and monitoring ILD.

Question	Consensus (*n* = 14)
1. Should patients with newly diagnosed RA be referred for mandatory ILD screening?	Patients should be referred for screening (*n* = 14)
2. What is the most important clinical symptom in newly diagnosed RA patients indicating to ILD?	Exertional dyspnea (*n* = 13) Persistent dry cough (*n* = 12)
3. What is the most important screening test for ILD in newly diagnosed RA patients?	Pulmonary function tests (*n* = 14)
4. What are the most relevant pulmonary function test results for ILD screening?	DLco < 80% of predictive value (*n* = 14) FVC < 80% of predictive value (*n* = 13)
5. What test should be considered crucial for follow-up in RA patients with confirmed ILD?	Pulmonary function tests (*n* = 14) Clinical examination (*n* = 10) HRCT (*n* = 8)
6. What should be considered crucial for follow-up in RA patients without confirmed ILD?	Clinical monitoring (*n* = 13)
7. How often should chest X-ray be repeated in stable RA-ILD patients?	In case of symptom worsening (*n* = 9)
8. How often should pulmonary function tests be repeated in stable RA-ILD patients?	Every 6–12 months (*n* = 12)
9. How often should HRCT be repeated in stable RA-ILD patients?	In case of symptom worsening (*n* = 13)
10. How often should chest X-ray be repeated in stable RA patients without confirmed ILD?	In case of symptom worsening (*n* = 13)
11. How often should pulmonary function tests be repeated in stable RA patients without confirmed ILD?	In case of symptom worsening (*n* = 9) Every 12 months (*n* = 5)
12. How often should HRCT be repeated in stable RA patients without confirmed ILD?	In case of symptom worsening (*n* = 12)

Abbreviations: DLco, diffusion capacity for carbon monoxide; FVC, forced vital capacity; HRCT, high-resolution computed tomography; ILD, interstitial lung disease; RA, rheumatoid arthritis.

**Table 4:** Consensus results of the IIM questionnaire for screening and monitoring ILD.

Question	Consensus (*n* = 14)
1. Should patients with newly diagnosed IIM be referred for mandatory ILD screening?	Patients should be referred for screening (*n* = 13)
2. What is the most important clinical symptom in newly diagnosed IIM patients indicating to ILD?	Persistent dry cough (*n* = 12) Exertional dyspnea (*n* = 11)
3. What is the most important screening test for ILD in newly diagnosed IIM patients?	Pulmonary function tests (*n* = 14) Serological test (*n* = 13) Clinical examination (*n* = 11)
4. What are the most relevant pulmonary function test results for ILD screening?	DLco < 80% of predictive value (*n* = 14) FVC < 80% of predictive value (*n* = 13)
5. What test should be considered crucial for follow-up in IIM patients with confirmed ILD?	Pulmonary function tests (*n* = 14) Clinical examination (*n* = 10) HRCT (*n* = 8)
6. What should be considered crucial for follow-up in IIM patients without confirmed ILD?	Pulmonary function tests (*n* = 13) Clinical monitoring (*n* = 11)
7. How often should chest X-ray be repeated in stable IIM-ILD patients?	In case of symptom worsening (*n* = 9)
8. How often should pulmonary function tests be repeated in stable IIM-ILD patients?	Every 6–12 months (*n* = 14)
9. How often should HRCT be repeated in stable IIM-ILD patients?	In case of symptom worsening (*n* = 13)
10. How often should chest X-ray be repeated in stable IIM patients without confirmed ILD?	In case of symptom worsening (*n* = 13) Every 12 months (*n* = 3)
11. How often should pulmonary function tests be repeated in stable IIM patients without confirmed ILD?	Every 12 months (*n* = 10) In case of symptom worsening (*n* = 3)
12. How often should HRCT be repeated in stable IIM patients without confirmed ILD?	In case of symptom worsening (*n* = 13)

Abbreviations: DLco, diffusion capacity for carbon monoxide; FVC, forced vital capacity; HRCT, high-resolution computed tomography; IIM, idiopathic inflammatory myopathy; ILD, interstitial lung disease.

**Table 5:** Susceptibility genes, autoantibodies, and serological immune markers associated with CTD-ILD.

Disease	Susceptibility Genes	Autoantibodies and Serological Immune Markers
RA-ILD [10,11,12,13,14,15,16]	DRB1 * 16:02, DRB1 * 15:02 TERT, RTEL1, PARN or SFTPC MUC5B	RF anti-CCP
SSc-ILD [10,17,18,19,20,21,22,23,24,25,26,27,28,29]	HLA-B * 62, HLA-C * 06, DRB1 * 11 DPB1 * 03:01, DR51 CD226, MMP12, SFTPB, CTGF, HGF, IRAK1, TCRBV, IRF5 CD247	anti-Scl-70 anti-U3RNP anti-U11/U12RNP anti-RuvBL1/2 anti-EIF2B anti-PM-Scl anti-U1RNP anti-cardiolipin anti-Th/To anti-Ro52 anti-NOR90 nucleolar ANA ANCA
PM/DM-ILD [10,30,31]	DRB1 * 03, DRB1 * 01:01, DRB1 * 04:05 DQB1 * 06:02	MSAs anti-Jo-1 anti-PL-12 anti-PL-7 anti-KS anti-OJ anti-EJ anti-Zo anti-Ku anti-MDA5 MAAs anti-Ro52/60 anti-U1RNP
MCTD-ILD [10,32]	TERC, TERT	Anti-U1RNP CIC C3 CH50
pSS-ILD [10]		anti-SSA/Ro anti-SSB/La
SLE-ILD [33]		anti-La anti-Scl-70 anti-U1RNP

ANCA, anti-neutrophil cytoplasmic antibodies; Anti-CCP, anti-citrullinated peptide antibodies; CD, clusters of differentiation; CIC, circulation immunity compound; CTD, connective tissue disease; CTGF, connective-tissue growth factor; DM, dermatomyositis; HGF, hepatocyte growth factor; HLA, Human Leukocyte Antigen; ILD, interstitial lung disease; IRAK, IL-1 receptor-associated kinase; IRF5, recombinant interferon regulatory factor 5; MAAs, myositis-associated antibodies; MDA5, melanoma differentiation-associated gene 5; MCTD, mixed connective tissue disease; MMP, matrix metalloproteinase; MSAs, myositis-specific autoantibodies; MUC5B, recombinant Mucin 5 Subtype B; PARN, polyadenylation-specific ribonuclease deadenylation nuclease; PM, polymyositis; pSS, primary Sjögren’s syndrome; RA, rheumatoid arthritis; RF, rheumatoid factor; RTEL1, telomere-elongation helicase-1; SFTPB, surfactant protein B; SFTPC, surfactant protein C; SLE, systemic lupus erythematosus; SSc, systemic sclerosis; TCRBV, T-cell receptor-b variable; TERC, telomerase RNA component; TERT, telomerase reverse transcriptase.

## Data Availability

Data are available from the corresponding author contact upon request.
